# Adolescent Nutrition—Developing a Research Agenda for the Second Window of Opportunity in Indonesia

**DOI:** 10.1177/0379572120983668

**Published:** 2021-07-20

**Authors:** Robert Sparrow, Rina Agustina, Hilde Bras, Grace Sheila, Matthias Rieger, Athia Yumna, Edith Feskens, Alida Melse-Boonstra

**Affiliations:** 1 Development Economics Group, Wageningen University, the Netherlands; 2 International Institute of Social Studies, Erasmus University Rotterdam, the Netherlands; 3 Department of Nutrition, Faculty of Medicine, Universitas Indonesia - Dr. Cipto Mangunkusumo General Hospital, Jakarta, Indonesia; 4 Human Nutrition Research Center, Indonesian Medical Education and Research Institute (IMERI), Faculty of Medicine, Universitas Indonesia, Jakarta, Indonesia; 5 Economic and Social History, 3647 University of Groningen, the Netherlands; 6 The 391969 SMERU Research Institute, Jakarta, Indonesia; 7 Division of Human Nutrition and Health, Wageningen University, the Netherlands

**Keywords:** adolescents, nutrition, Indonesia, research agenda

## Abstract

**Background:**

Recently, adolescence has been identified as a second window of opportunity for the correction of nutritional inadequacies. However, there is a lack of knowledge on evidence-based integrated nutrition strategies for adolescents in Indonesia.

**Objective:**

To provide a research agenda and the prioritization of research actions to tackle outstanding knowledge gaps on adolescent nutrition in Indonesia.

**Methods:**

A preliminary set of research topics was listed based on a desk study of the academic literature and policy documents. Second, a stakeholder meeting was held to further identify and discuss research topics related to adolescent nutrition in Indonesia. Third, an online survey was conducted in which respondents were asked to indicate priority research themes for the next 3 to 5 years and to rank a total of 23 research questions.

**Results:**

Most (52%) of the respondents who returned the survey (n = 27) prioritize research on implementation and program evaluation, while 30% prefer descriptive and explanatory research, and 19% place priority with intervention and discovery research. However, when we followed up with specific topics for each of these broad research areas, a more nuanced picture emerged, with intervention and discovery research taking a more prominent standing.

**Conclusions:**

In order to support the design, implementation, and effectiveness of integrated nutrition programs for Indonesian adolescents, in-depth studies should question the best intervention strategies, modes of delivery, and long-term outcomes, while nationwide and disaggregated data should investigate associations and trends over time and identify vulnerable groups.

## Introduction

Sustainable Development Goal no. 2, “Zero Hunger,” aims to ensure universal access to safe, nutritious, and sufficient food all year round by the year 2030 and specifically includes the target of addressing the nutritional needs of adolescent girls. Adolescents and youth, particularly girls and young women, are increasingly seen as driving forces for global health and international development. Investments in the capabilities of the world’s 1.2 billion adolescents of 10 to 19 years of age are considered to be vital to the United Nations’ (UN) Sustainable Development Agenda, yielding high social and economic returns.^1^

Adolescence is characterized by profound physical growth and cognitive and socioemotional development, which are heavily influenced by an individual’s social, economic, and cultural environment.^2^ Adequate nutrition during this critical age period is associated with the improved health and development of the future adult population, as well as that of their future offspring, bringing potential intergenerational benefits.^3^ Following the first window of opportunity to correct malnutrition in the first 1000 days of life, adolescence has been identified as a second window of opportunity for the correction of nutritional inadequacies and insufficient growth from childhood.^4^

Indonesia is undergoing a rapid nutrition transition, resulting in a triple burden of malnutrition, with persistent stunting and anemia on the one hand and increased overweight and noncommunicable diseases on the other. Dietary risks are the leading contributors to the burden of disability-adjusted life-years (DALYs) in Indonesia, accounting for 13.6% of DALYs in 2016.^5^ Adolescence is an important period in life for the internalization of long-term lifestyle habits. Adolescent nutrition has, however, generally been a neglected area both in research and policy nationally as well as globally. Today’s adolescents are more exposed to nutritional risks, harmful behavior, lack of physical activity, smoking and substance use, sexually transmitted diseases, and other risks than in the past and face new challenges in a changing technological context.^6,7^

With the onset of a nutrition transition in Indonesia, an appropriate research agenda needs to be set out to guide effective interventions to address adolescent nutrition. To do this, the aim of this article is to provide a research agenda by prioritizing research actions to tackle the outstanding knowledge gaps in Indonesia. As outlined in the next section, the research agenda is anchored in a set of interlinked trajectories that, together, define an adolescent’s transition to adulthood. The subsequent section then discusses the research themes, topics, and questions that follow. Finally, based on stakeholder engagement (through a stakeholder workshop, focus group discussions, and an online survey), we translate these research questions into a specific research agenda for Indonesia.

## Transition to Adulthood: Interlinked Trajectories

**Figure 1.**
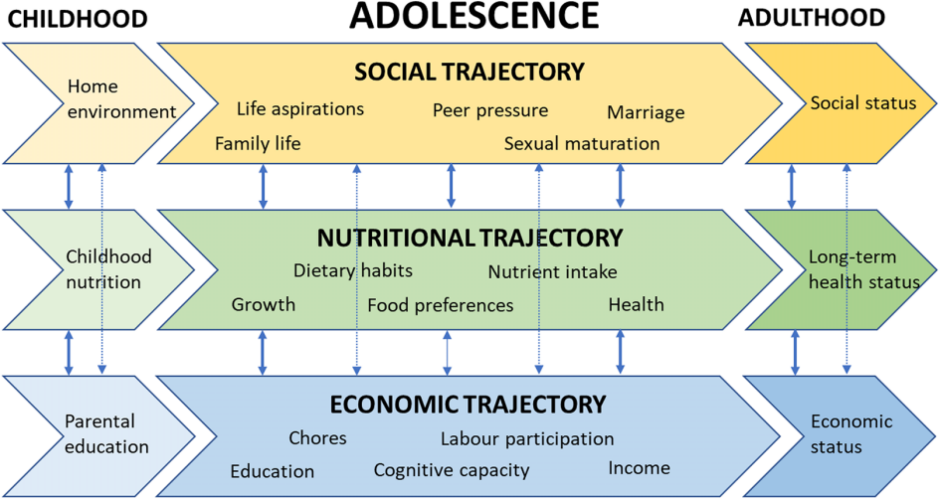
An impression of the intricate relationships between the social, economic, and nutritional trajectories of adolescents in a life course perspective.

During the transition to adulthood, the nutritional trajectories (eg, nutritional status, dietary intake) of adolescents are interwoven with social and economic trajectories, including education, family formation, and labor participation, which sets them apart from younger children and adults (Figure 1). These trajectories are influenced by the household and immediate context, including that of the food system and sociocultural setting.^8^ Understanding of the interconnections between these trajectories is crucial when setting a research agenda for adolescent nutrition.

The “nutrition trajectories” refer to the increased nutrient requirements during the transformation from puberty to adulthood and parenthood, which in girls is reflected by a gain in pelvic width, mammary and uterine tissue, and adipose tissue and depends more on sexual maturation than chronological age.^7^ The main landmarks of female pubertal development are the visible start of breast development, indicating the onset of the growth spurt (occurring at 10 to 14 years of age), and the associated first menstrual bleeding: the menarche, occurring at 11 to 16 years of age.^9-11^ Girls who live under less favorable circumstances generally experience menarche at a later age.^12^ Age at menarche (AAM) is an important landmark in adolescent girls, both nutritionally and socially.^10,11^ Most height is gained 1 to 1.5 years before menarche, but additional height and the growth of pelvic bones, critical for the prevention of obstructed labor, is gained just before menarche and over a period of 4.7 years after menarche.^13,14^ Up to 20% of total height and 45% of bone mass are achieved during adolescence, with a peak ponderal growth velocity that nearly matches growth during infancy; therefore, this life period may offer the possibility for catch-up growth from malnutrition suffered during childhood. Age at menarche is not only associated with early life factors but also reflects future health.^15^ Early menarche (before 11 years of age) increases the risk of abdominal type obesity, glucose intolerance and insulin resistance, cardiovascular risk, coronary heart disease, and increased cancer mortality (especially breast cancer). Women with earlier menarche also attain a shorter height than women with a normal AAM.^16,17^ Early menarche in regions where fertility is high, especially in low- and middle-income countries (LMICs), may be associated with pregnancy in adolescence. Teenage pregnancy puts female adolescents at a greater disadvantage due to the increased nutritional requirements brought about by pregnancy on top of their own requirements, resulting in slowing and stunting a girl’s growth and leading to a higher risk of complications and mortality for mother and child and poorer birth outcomes.^18-20^ Late menarche (above 16 years of age), on the other hand, increases the risk of osteoporosis, adolescent depression, and symptoms of social anxiety.^21^

It is generally recognized that optimizing the nutritional status of adolescent girls is essential for their own health, as well as that of their offspring. There are, however, many knowledge gaps in adolescent nutrition. There is a general lack of information on dietary practices, nutrient intakes and behaviors, and a key evidence gap on the optimal timing (before or after AAM) for nutrition interventions.^22-24^ Of interest is also the possible competition for nutrients between the pregnant adolescent and her fetus. Birth weight may be compromised when the adolescent growth spurt continues during pregnancy. Early pregnancies are also assumed to affect the growth of adolescents themselves,^17,25,26^ but the mechanism is not fully understood.^27^ In view of the different growth patterns of boys during pubertal development, it is unknown whether boys would benefit as much as girls from nutritional interventions, although good nutrition for boys is an important goal on its own. A recent systematic review on evidence-based adolescent nutrition interventions underscored the importance of improving nutrition, combined with other interventions during this critical age. As yet, there is a paucity of trials from LMICs.^28^

“Social trajectories” refer to the pathways which adolescents follow throughout their education and family formation (eg, marriage and pregnancies). The most immediate social context for most individuals, including adolescents, is the family or household where, due to the status of the adolescents, inequalities may exist in the allocation of resources to adolescents according to their age, gender, or birth order, depriving them of necessary foods and nutrients.^29,30^ Cultural norms and practices often lie at the base of such inequalities, and gender, in combination with age and birth order, seems to be important in determining children’s and youngsters’ nutritional status, with earlier born children and boys found to be favored in many Asian regions.^31-34^

Recent research, using a life course perspective, increasingly shows the linkages between infant and childhood disadvantage and adolescent and young adult outcomes. Existing research has established that poor nutritional status at age 3 is linked to lower grade attainment and poorer cognitive skills in adulthood.^35^ Poor health and nutrition impact children’s educational attainment because they reduce their time in school and their learning during that time.^36^ The long-term effects of socioenvironmental factors on improved cognition are highlighted by Prado et al, suggesting that interventions focused on biomedical determinants need to be complemented by programs which address socioenvironmental determinants.^37^ A longitudinal study in 4 LMICs also showed a link between malnutrition and noncognitive skills. A higher height-for-age at the age of 7 to 8 was associated with increased self-efficacy, self-esteem, and aspirations at the age of 11 to 12.^38,39^ The causality of these associations has not been proven, however, and may well result from common root causes that pertain throughout infancy and childhood.

Another important linkage that needs further investigation is that between nutrition, child marriage, and pregnancy. Globally, 1 in 3 girls in LMICs across the world marries before the age of 18.^40,41^ Research shows that poverty and food insecurity in the parental home are among the main causes of early marriage. For many poor parents, marrying off a daughter at an early age means one mouth less to feed.^42-44^ Child marriage and pregnancy, in turn, directly impact the health of these girls and that of their offspring. Complications from pregnancy and childbirth are the main cause of death among adolescent girls aged 15 to 19 in LMICs.^40^ Stillbirths and newborn deaths are 50% higher among the infants of adolescent mothers than they are among mothers between the ages of 20 and 29.^40^ Moreover, girls who marry too young do not have the time to be educated, to mature, and to develop their self-esteem and their status in their households, which would allow them to protect their and their children’s nutritional status.^41^

It is still largely unknown, however, whether and how nutrition in adolescence, as a second window of opportunity, affects young adult outcomes in education and marriage and entry into parenthood at a (too) early age. The question is how nutrition and socioeconomic outcomes mutually influence each other and whether the so-called “bundles of disadvantage” are determined by the same factors, such as gender and birth order.^45^ In addition, depending on their status in the family or household, adolescents may be important sources of social influence and act as change agents toward healthy diets.

“Economic trajectories” focus on labor market participation and the contribution adolescents may make to the (in)formal household labor force. In general, however, the contribution of adolescents to the household economy is not made visible. The size of this contribution and its potential multiplier effects may depend on factors such as the relative scarcity of labor in family businesses, wage rates for adolescents in the labor market, the (economic) value of education, and the adolescent’s gender. In brawn-based developing economies, improved nutrition may especially improve the productivity and wage rates of men and, to a lesser extent, those of women.^46^ It is unknown how this translates into gendered labor force participation and the wage rates/productivity of adolescents and, thus, whether improved nutrition and health status contribute to the higher economic value of adolescents.

In contrast, intrahousehold resource allocation is often based on the “contribution role,” indicating that the person(s) contributing most to labor or the income of the household receive more resources.^29,30^ An increased contribution to food supply or income may result in more favorable intrahousehold resource allocation to adolescents depending on a myriad of factors, including the gender of the adolescent and the importance of marriage and a dowry. Information on the economic returns to various types of investment in youth development is scarce, and these returns are likely to be positively correlated with investments during early childhood. The above-described potential effects of nutrition on the education of adolescents will be mediated through economic considerations, as there may be trade-offs between education, labor, and marriage trajectories. Higher wages associated with better nutrition may drive male adolescents out of school earlier than female adolescents, thus increasing the schooling of women compared to that of men, thereby leading to occupational differentiation by gender.^46^ The tradition of the bride price may augment the positive effect of female education on improved nutrition, as higher prices are paid for better educated girls.^47^ In addition, healthier (male) adolescents from rural areas have better opportunities of finding urban jobs and may thus be more likely to migrate. On the other hand, education may also facilitate migration opportunities in the long run.^48^ While the choice between education, local wage employment, and migration at adolescence is key to earning capacity at adulthood, these potential mechanisms and their consequences have so far received very little empirical substantiation.

In addition to education and working experience, personal preferences have a large influence on economic decision-making. Adolescence is a crucial period in the development of the brain and is especially important for the development of personal traits such as social preferences, patience, and risk aversion.^49^ (Mal)nutrition at adolescence may therefore have persistent effects on economic decisions in later life. Thus far, research on the biological determinants of preferences has largely focused on hormonal fluctuations (in utero and during the menstrual cycle), neglecting the potentially important role of nutrition.

These seemingly parallel nutrition, social, and economic trajectories are often interlinked, and changes in, for example, the nutrition trajectory may have synergies as well as trade-offs in the other two trajectories related to health, education, family formation, and labor participation with different consequences across the life course. For instance, early marriage may be followed by early pregnancy, which in turn leads to nutritional stress before completion of a girl’s own physical growth, reducing her chances of education and labor.^50^ Household culture (such as deeply rooted food habits) and composition (such as household size, including sibling status, and family type) may affect nutritional status.^51^ In turn, poor nutritional status is associated with noncognitive skills, psychosocial competencies, perceptions, and future aspirations. Moreover, the mechanisms underlying these links appear to be highly gendered, with important gender gaps in the link between nutrition, aspirations, and education and labor market outcomes.^52^

In summary, increased attention to adolescent nutrition and health is needed to reverse deficiencies that occurred earlier in life, as well as to foster a healthy transition from childhood to adulthood. This should set the stage for the attainment of a sufficient educational level, earning capacity, and sound family and partner relations to enable them to protect their own and their offspring’s nutrition status and prevent or delay adult-onset diet-related illnesses. A life course perspective offers the opportunity to study the multiple pathways in the nutrition, economic, and social domains and their interaction and interrelationships (Figure 1). This will provide a better understanding of the importance of the period of adolescence and the type and efficacy of nutrition interventions needed for this age group, as well as the social and economic consequences of such interventions for the health and development of this age group and their offspring, now and later in life.

## International Research Priorities for Adolescent Nutrition

Due to the interlinkages between the nutrition and social and economic trajectories, it is imperative to combine nutrition-*specific* interventions (ie, micronutrient supplementation, nutrition education, school feeding programs) with nutrition-*sensitive* interventions addressing the root causes indirectly related to malnutrition (ie, poverty reduction programs, basic education, reproductive health literacy, gender inequalities, and the health system environment). This requires research programs that address current knowledge gaps from 3 different angles: (1) descriptive and explanatory research to identify population groups at risk by making use of (sub)nationally representative survey data, (2) intervention and discovery research to develop new strategies and provide proof-of-principle of cause and effect, and (3) implementation and program evaluation research to learn what platforms can be used and what works or does not work for this age group.

Internationally, several topics have been identified for further investigation,^3,4,28,53,54^ including:

- detailed and representative dietary intake data of adolescents and a cross-cultural validated dietary quality index for adolescents;
- the impact of adolescent nutritional practices on subsequent pregnancies, pregnancy outcomes, and offspring health;
- better understanding of the psychosocial drivers of food choice (eg, imprinted eating behavior, taste preferences, body image, peer pressure, and their relation to the physiology of sleep, physical activity, puberty, growth spurt, and adult health);
- better understanding of the economic drivers of food choice (eg, convenience, price, marketing, and the changing food environment);
- evidence on the effectiveness of scalable nutrition-specific interventions (eg, iron–folic acid supplementation and enriched food products); and
- evidence on the effectiveness of scalable nutrition-sensitive interventions (eg, social safety nets, the inclusion of nutrition in adolescent reproductive health programs).

## Formulating a Research Agenda for Indonesia

Indonesia is home to approximately 24 million adolescent girls. According to national basic health survey (RISKESDAS) data collected in 2010, 2013, and 2018, there is little progress in the reduction of stunting and thinness among adolescents, whereas the prevalence of overweight and obesity has increased.^55-57^ Moreover, an increase in the prevalence of anemia has been observed over the last decade, showing that 84.6% of pregnant girls aged 15 to 24 years were anemic in 2018. A supplementation program with iron–folic acid capsules targeting adolescent girls aged 12 to 18 years began in 2016 and is currently being rolled out across the country. However, the diet quality of adolescents in Indonesia is low, according to the latest Total Diet Study (2014).^58^ Adolescents aged 13 to 18 years have the lowest protein adequacy, with the age-specific recommendation not being met by 33.5%. In addition, they consume an average daily amount of vegetables and fruits, which is only 13% to 15% of what is being recommended. On the other hand, 30% exceed the upper limit for fat intake and 26% exceed the upper limit for sodium intake.

The age-specific fertility rate per 1000 adolescent girls aged 15 to 19 has come down from 48 in 2012 to 36 in 2017, with 7% of 15- to 19-year-old girls being pregnant or already having given birth to a child in 2017.^59^ Pregnancy and lactation pose additional nutritional stress on an adolescent girl’s growing body, which forms a risk factor not only for her own health but also for that of her child. It was estimated that approximately 38.5% of pregnant girls aged 15 to 19 experience chronic energy deficit.^56^ Malnutrition in adolescent girls is therefore a major determinant of low birth weight and stunting in babies, as well as a risk factor for complications during childbirth and perinatal mortality. In addition to national surveys, some academic research has been done on adolescent nutrition in Indonesia, but published data are scant.

In a comparison including 57 LMICs, Indonesia ranks one of the countries with the highest prevalence of stunting and thinness among adolescents aged 12 to 15, respectively. Conversely, Indonesia still ranks low in terms of the prevalence of overweight and obesity in comparison with other countries in this age group.^60^ This suggests that Indonesia’s nutrition transition is at a relatively early stage, leaving scope for a timely policy response to relieve some of its more severe symptoms. But, as yet, a research agenda that addresses the specific Indonesian context regarding adolescent nutrition has not been developed. Our objective is therefore to formulate a research agenda for adolescent nutrition in Indonesia based on extensive stakeholder engagement.

### Listing, Selection, and Prioritization of Research Topics

The exercise involved 3 steps. First, we identified a preliminary set of research topics and questions, based on a desk study of the academic literature and policy documents for Indonesia.

Second, a stakeholder meeting was held on adolescent nutrition in December 2018 in order to (1) raise awareness on the topic, (2) take stock of the current research landscape, (3) identify research gaps in support of adolescent nutrition policy and programming, and (4) create partnerships for research and program evaluation. The meeting was attended by 102 participants representing a broad range of stakeholders from government (the Presidential Staff Office, Ministry of Health, Ministry of Education and Culture, Ministry of National Development Planning, Ministry of Religious Affairs, Agency for the Assessment and Application of Technology [BPPT], National Population and Family Planning Board, and TNP2K), academia (Universitas Indonesia, the Center for Indonesian Medical Students’ Activities, the Indonesia International Institute for Life-Sciences, the Southeast Asian Ministers of Education Regional Centre for Food and Nutrition, Universitas Sahid, Wageningen University & Research, and ISS Erasmus), UN/nongovernmental agencies (the United Nations Children’s Fund, the United Nations Population Fund, SMERU, the World Food Programme, the World Bank, GAIN, Helen Keller International, Aliansi Remaja Independen, Nutrition International, the Indonesian Nutrition Society (PERSAGI), Foodbank of Indonesia, and the Center for Indonesia’s Strategic Development Initiatives), and the private sector (Indofood, Unilever, Frisian Flag, the Indonesian Food & Beverage Association [GAPMMI], Choices International Foundation, and Japfa Comfeed).

During the stakeholder meeting, a number of topics relating to adolescent nutrition in Indonesia were identified as important and requiring further research. Moreover, the preliminary research topics and questions were addressed in plenary presentations and focus group discussions.

Third, we conducted an online survey to assess the relevance of the research questions among the stakeholders who attended the workshop (n = 92). The research questions were refined based on the input from the stakeholder meeting. The survey was conducted in May to June 2019 and it took respondents 20 to 30 minutes to complete the set of 10 questions. The first question concerned the type of research respondents would prioritize. The survey then asked respondents to indicate the level of priority of research themes for the next 3 to 5 years. This also involved the ranking of 23 research questions based on the stakeholder workshop. The questions were categorized by (1) descriptive and explanatory research, (2) intervention and discovery research, and (3) implementation and program evaluation research. The survey ended with a question about the sector in which respondents were actively engaged.

### Results of the Survey

The survey saw a 29% response rate (n = 27): 70% from academia, 11% from government, and 19% from civil society and nongovernmental organizations. We first asked a set of general questions as to what research on adolescent nutrition deserved the highest priority. Over half (51%) of the respondents prioritized research on implementation and program evaluation concerning adolescent nutrition in Indonesia, while 30% preferred descriptive and explanatory research and 19% placed priority with intervention and discovery research. However, when we followed up with specific topics for each of these broad research areas, a more nuanced picture emerged, with intervention and discovery research taking a more prominent standing.

Table 1 reports the ranking of all the research questions categorized according to (1) descriptive and explanatory research, (2) intervention and discovery research, and (3) implementation and program evaluation research. The top 10 prioritized research topics for Indonesian adolescents by the respondents were:

1. the long-term impact of adolescent nutrition on their health as an adult (eg, metabolic syndrome, bone health);
2. the effectiveness of behavior change communication programs on nutritional status;
3. insight into the etiology of anemia in adolescent boys and girls (eg, iron deficiency, other nutritional factors, infectious diseases, genetics);
4. the effectiveness of multi-micronutrients versus iron/folic acid supplementation;
5. eating preferences and how to influence these;
6. the role of the sociocultural environment (eg, ethnicity, urban/rural, family structure, gender) on dietary intake and nutritional status;
7. insight into physical (in)activity patterns and how to change these to curb the increase in overweight/obesity;
8. developing a dietary quality index for Indonesian adolescents;
9. insight into the link between nutrition and behavioral/mental disorders and self-esteem (eg, body image, peer pressure, well-being, depression); and
10. how to create sustained healthy eating trends (eg, using social media, role models, chefs, peer-to-peer education).

**Table 1.** Ranking of Research Topics.

Overall rank	Descriptive and explanatory research
6	Role of the sociocultural environment (eg, ethnicity, urban/rural, family structure) on dietary intake and nutritional status
9	Insight into the link between nutrition and behavioral/mental disorders and self-esteem (eg, body image, peer pressure, well-being, depression)
11	Nationally representative time trends in nutritional status and its determinants
14	Impact of social media use on nutrition behavior and outcomes
18	Effects of migration within Indonesia on dietary patterns
19	Effects of economic and social globalization on dietary patterns and nutritional status
20	Long-term impact of nutritional status during adolescence on socioeconomic outcomes
21	Relationship between nutritional status and pubertal onset and vice versa
Rank	Intervention and discovery research
1	Long-term impact of adolescent nutrition on health as an adult (eg, metabolic syndrome, bone health)
3	Insight into the etiology of anemia in adolescent boys and girls (eg, iron deficiency, other nutritional factors, infectious diseases, genetics)
5	Eating preferences and how to influence these
7	Insight into physical (in)activity patterns and how to change these to curb increase in overweight/obesity
8	Developing a dietary quality index for Indonesian adolescents
15	Effect of nutritional interventions on life aspirations, self-esteem, and girls’ agency
16	Role of nutrition during adolescence on brain development
17	Timing of interventions during adolescence to improve linear growth
21	Effect of nutritional interventions on education, age at marriage, and labor participation
Rank	Implementation and program evaluation research
2	Effectiveness of behavior change communication programs on nutritional status
4	Effectiveness of multi-micronutrients vs iron/folic acid supplementation
10	How to create sustained healthy eating trends (eg, using social media, role models, chefs, peer-to-peer education)
12	How to shape a healthy food environment in and around schools
13	Evaluation of iron/folic acid supplementation program
21	Implementation and evaluation of a nutritional safety net program for adolescents living in the poorest families

## Conclusion

Indonesia is currently on the verge of undergoing a major nutrition transition, which can be expected to result in a rapid increase of overnutrition, on top of unresolved undernutrition and micronutrient deficiencies. Investing in adolescent nutrition is one of the cornerstones of creating a healthy generation. Due to the interrelationships between the nutritional, social, and economic trajectories during adolescence, nutrition-specific interventions alone will not be sufficient to curb the looming consequences of the nutrition transition. Integrated nutrition programs coupled with behavior change aimed at shifting adolescents away from adverse dietary habits are urgently required.

The current knowledge gaps in the nutritional situation of adolescents living in Indonesia prohibit evidence-based action, although some information can be extrapolated from younger children and older women. But, unless the interactions between nutrition, social, and economic life trajectories within the Indonesian context are well understood, intervention programs aiming to improve public health nutrition during adolescence are doomed to fail. The research agenda presented here supports the need to fill the knowledge gaps in the current understanding of the nutrition situation of Indonesian adolescents. While investment in integrated nutrition programs for this age group is warranted, in-depth studies should question the best intervention strategies, modes of delivery, and long-term outcomes. In parallel, nationwide and disaggregated data should be analyzed to investigate associations and trends over time and identify vulnerable groups. We hope that this research agenda will be of help to academia, governmental and nongovernmental workers, and private sector to prioritize and align their research activities into a collective effort to address adolescent nutrition. The next generation of Indonesian adolescents should be able to nourish themselves healthily, without placing a burden on their physical, social, and economic development.
